# Burden of chronic kidney diseases and underlying causes in Zambia: evidence from the global burden of disease study 2019

**DOI:** 10.1186/s12882-023-03078-5

**Published:** 2023-02-18

**Authors:** Samuel Bosomprah, Erica C. Bjonstad, John Musuku, Namasiku Siyumbwa, Mwila Ngandu, Mukobe Chisunka, Patrick Banda, Fastone Goma, Aggrey Mweemba

**Affiliations:** 1 Research Department, Centre for Infectious Diseases Research in Zambia, Lusaka, Zambia; 2 Department of Biostatistics, School of Public Health, University of Ghana, P.O. Box LG 13, Legon Accra, Ghana; 3 Noncommunicable Diseases and Injury Commission, Lusaka, Zambia; 4 Department of Pediatrics, University of Alabama at Birmingham, Birmingham, USA; 5 Ministry of Health, Lusaka, Zambia; 6 Department of Internal Medicine, University Teaching Hospital, Lusaka, Zambia; 7 Department of Internal Medicine, Levy Mwanawasa University Teaching Hospital, Lusaka, Zambia

**Keywords:** Chronic kidney disease, Hypertension, Diabetes, Disease burden, Underlying causes

## Abstract

**Introduction:**

Chronic kidney disease (CKD) has been a global public health problem and a major source of suffering and poor quality of life for those afflicted. Using data from the global burden of disease (GBD) study 2019, we estimated the magnitude of the burden of CKD as well as the underlying causes of CKD in the Zambian population.

**Method:**

The data used for this study were extracted from the GBD 2019 study. The GBD 2019 provides estimates of several metrics of disease burden including the commonly used disability-adjusted life year (DALYs) for over 369 diseases and injuries, and 87 risk factors and combinations of these in 204 countries and territories from 1990 to 2019. We estimated the burden of CKD as the number and rates (per 100,000 population) of DALYs, disaggregated by year, sex, and age group. We examined the underlying causes of CKD by estimating the population attributable fraction as the percentage contributions of risk factors to CKD DALY.

**Results:**

The number of DALYs for CKD was estimated as 76.03 million (95% UI: 61.01 to 93.36) in 2019 compared to 39.42 million (95% UI: 33.09 to 45.90) in 1990, representing 93% increase whereas the DALYs rate per 100,000 population was estimated as 416.89 (95% UI: 334.53 to 511.93) in 2019 compared to 496.38 (95% UI: 416.55 to 577.87) in 1990, representing 16% reduction. CKD due to hypertension accounted for 18.7% of CKD DALYs and CKD due to diabetes (types 1 and 2) accounted for 22.7%, while CKD from glomerulonephritis accounted for the most DALYs at 33%. The age group most impacted from CKD were adolescents and young adults.

**Conclusion:**

The burden of CKD remains high in the Zambian population with diabetes, high blood pressure, and glomerulonephritis as important causes. The results highlight the need to develop a comprehensive action plan to prevent and treat kidney disease. Increasing the awareness of CKD among the public as well as adaptation of guidelines for treating patients with end stage kidney disease are important considerations.

## Introduction

Chronic kidney disease (CKD), a condition in which the kidneys are damaged and cannot filter blood as well as they should [1], has been a global public health problem. In 2017, there were an estimated 1·2 million (95% uncertainty interval [UI] 1·2 to 1·3) deaths from CKD [2, 3] globally. Between 1990 and 2017, there was approximately 41·5% (95% UI 35·2 to 46·5) increase in CKD-specific all-age mortality [2]. In 2017, about 697·5 million (95% UI 649·2 to 752·0) cases of all-stage CKD were recorded with a global prevalence of 9·1% (8·5 to 9·8). Globally, all-age prevalence of CKD increased by approximately 29·3% (95% UI 26·4 to 32·6) since 1990. The burden of CKD was much higher in several regions including, Oceania, sub-Saharan Africa (SSA), and Latin America [2].

CKD is a major source of suffering and poor quality of life. They are associated with multiple adverse outcomes, including increased risk of death, progression to end-stage kidney disease (ESKD) (requiring dialysis or kidney transplantation), and increased risk of other co-morbid diseases like hypertension and cardiovascular diseases. In a prospective cohort study of people with CKD stage 3, Shardlow and colleagues [4] found that about 14.2% of the patients had died, mostly due to cardiovascular causes, after five years of follow up. During the COVID-19 pandemic, it also became apparent that CKD was both a risk factor for worse outcomes as well as an unexpected potential outcome of any degree of COVID-19 infection [5, 6]. The rising burden of CKD in lower-resourced regions demands attention as its treatment (e.g., dialysis, transplantation) is often unattainable by the majority afflicted. Zambia, a lower-middle income country, does not have a kidney transplant program and less than 10% of ESKD adult patients can access chronic dialysis services. In a recent survey, there is limited application of international guidelines for CKD screening and prevention,[7] and Zambia has no national guidelines on CKD or its final phase ESKD.

Using data from the global burden of disease (GBD) study 2019, we aimed to estimate the magnitude of the burden of CKD as well as the average annual rate of change in the burden of CKD between 1990 and 2019 in Zambia. We also examined the underlying causes of CKD in the Zambian population. The results of this study can help stakeholders develop a comprehensive action plan to prevent and treat kidney disease. Though these results are specific to Zambia, their reach is important for the entire SSA sub-continent given the paucity of CKD data, particularly among children.

## Methods

### Conceptual framework and definitions

**Fig. 1:**
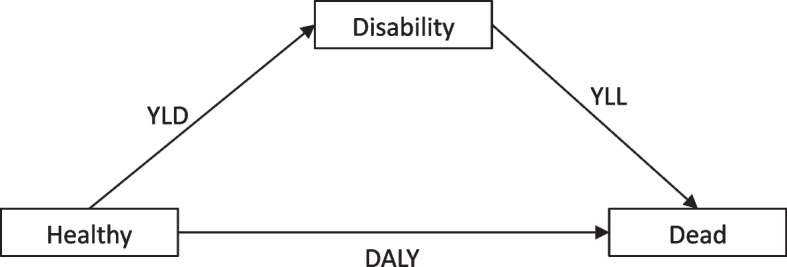
Healthy-Disability-Death Model; Source: First author’s conceptualization

The disability-adjusted life year (DALY) is the primary metric used to assess the global burden of disease [8]. One DALY can be thought of as one lost year of ‘healthy’ life and the measured disease burden is the gap between a population’s health status and that of a normative reference population [8]. Ranking the causes of DALY in a population shows the health problems that cause the most suffering in a society. DALYs for a specific cause are calculated as the sum of the years of life lost (YLL) from that cause and the years lived with disability (YLD) for people living in states of less than good health resulting from the specific cause (Fig. 1). The basic formula for DALY for given cause $c$, age $a$, sex $s$ and year $t$ is:

$$DALY\left(c,s,a,t\right)=YLL\left(c,s,a,t\right)+YLD\left(c,s,a,t\right)$$

The YLLs for a cause are essentially calculated as the number of cause-specific deaths multiplied by a loss function specifying the years lost for deaths as a function of the age at which death occurs [8]. It is a measure of premature death within a group of people. The basic formula for YLLs for a given cause $c$, age $a$, sex $s$ and year $t$ is:

$$YLL\left(c,s,a,t\right)=N\left(c,s,a,t\right)*L\left(s,a\right)$$

Where $N\left(c,s,a,t\right)$ is the number of deaths due to the cause $c$ for the given age $a$ and sex $s$ in year $t$ and $L\left(s,a\right)$ is a standard loss function specifying years of life lost for a death at age $a$ for sex $s$. The loss function is specified in terms of the life expectancies at various ages in standard life tables. For example, if achievable life expectancy in a given year for men is 65 years, a man who dies of lung cancer at age 46 will have 19 years of life lost. Life expectancy is the number of years that the average member of a group can expect to live.

The YLDs for a particular cause in a particular time period can be estimated as the number of incident cases in that period multiplied by the average duration of the disease and a weight factor that reflects the severity of the disease on a scale from 0 (perfect health) to 1 (dead) [8]. It measures the amount of time people lose to diseases and injuries that degrade health but do not cause death. This means that a short-term, severe health problem and a long-term, relatively mild health problem could both result in the same number of YLDs. For example, someone who needs two months to recover from a car accident but then regains their full health and someone who experiences relatively mild but lifelong back pain could end up losing the same number of years of their lives to disability. The basic formula for YLD for a given cause $c$, age $a$, sex $s$ and year $t$ is:

$$YLD\left(c,s,a,t\right)=I\left(c,s,a,t\right)*L\left(c,s,a,t\right)*W\left(c,s,a\right)=P\left(c,s,a,t\right)*W\left(c,s,a\right)$$

where $I\left(c,s,a,t\right)$ = number of incident cases for cause $c$, age $a$, sex $s$ and year $t$; $W\left(c,s,a\right)$ = disability weight for cause $c$, age $a$ and sex $s$ (ranges from 0= perfect health to 1=death); $L\left(c,s,a,t\right)$ = average duration of the case until remission or death (years); and $P\left(c,s,a,t\right)$ = number of prevalent cases for cause $c$, age $a$, sex $s$ and year $t$. The numerical valuation of time lived in non-fatal health states is through the so-called disability weights, which quantify loss of functioning on a scale where 0 represents perfect health and 1 represents a state equivalent to death. Depending on how these weights are derived and what they are attempting to quantify, they are variously referred to as disability weights, quality-adjusted life year (QALY) weights, health state valuations, utilities or health state preferences [8].

## Data sources

The data used for this study were extracted from the GBD study 2019 [9]. The GBD 2019 provides estimates of several metrics of disease burden including the commonly used DALYs for over 369 diseases and injuries, and 87 risk factors and combinations of these in 204 countries and territories from 1990 to 2019 [3, 10–12]. Detailed methods of GBD 2019 have been published elsewhere [11, 12]. The GBD 2019 attributable burden estimates followed the general framework established for comparative risk assessment (CRA) [13, 14].

Compared with earlier GBD studies [15–17], the GBD 2019 study updated its methodologies in estimating risk exposure and risk-attributable burden using data from several epidemiological studies in different countries [3, 11]. These studies included systematic reviews, clinical trials, cohort studies, case-control studies, and other observational studies [3, 11]. Our study adheres to the Guidelines for Accurate and Transparent Health Estimates Reporting (GATHER) recommendations [18]. Chronic kidney disease was classified as a disease as well as a metabolic risk factor in the GBD study [19], but it was considered as a disease in our analysis. CKD was defined as eGFR (estimated glomerular filtration rate) of < 60 mL/min per 1.73 m^2^ [19].

## Statistical analyses

We estimated the burden of CKD as the number and rates (per 100,000 population) of DALYs, disaggregated by year, sex, and age group. To examine the change in DALYs between 1990 and 2019, we calculated the arithmetic difference in DALYs between 2019 and 1990 expressed as a percent of 1990 DALYs. For each estimated rate and number of DALYs, we reported 95% uncertainty interval (UI), which was calculated by taking 1000 draws from the posterior distribution of each quantity and using the 25th and 975th ordered draw of the uncertainty distribution [20].

We used linear regression model with robust standard error to estimate the average annual rate of change in the burden of CKD since 1990. We examined the underlying causes of CKD by estimating the population attributable fraction as the percentage contributions of risk factors to CKD DALY. All analyses were performed using Stata 17 MP8 (StataCorp, College Station, TX, USA).

## Results

### Burden of chronic kidney diseases

**Fig. 2:**
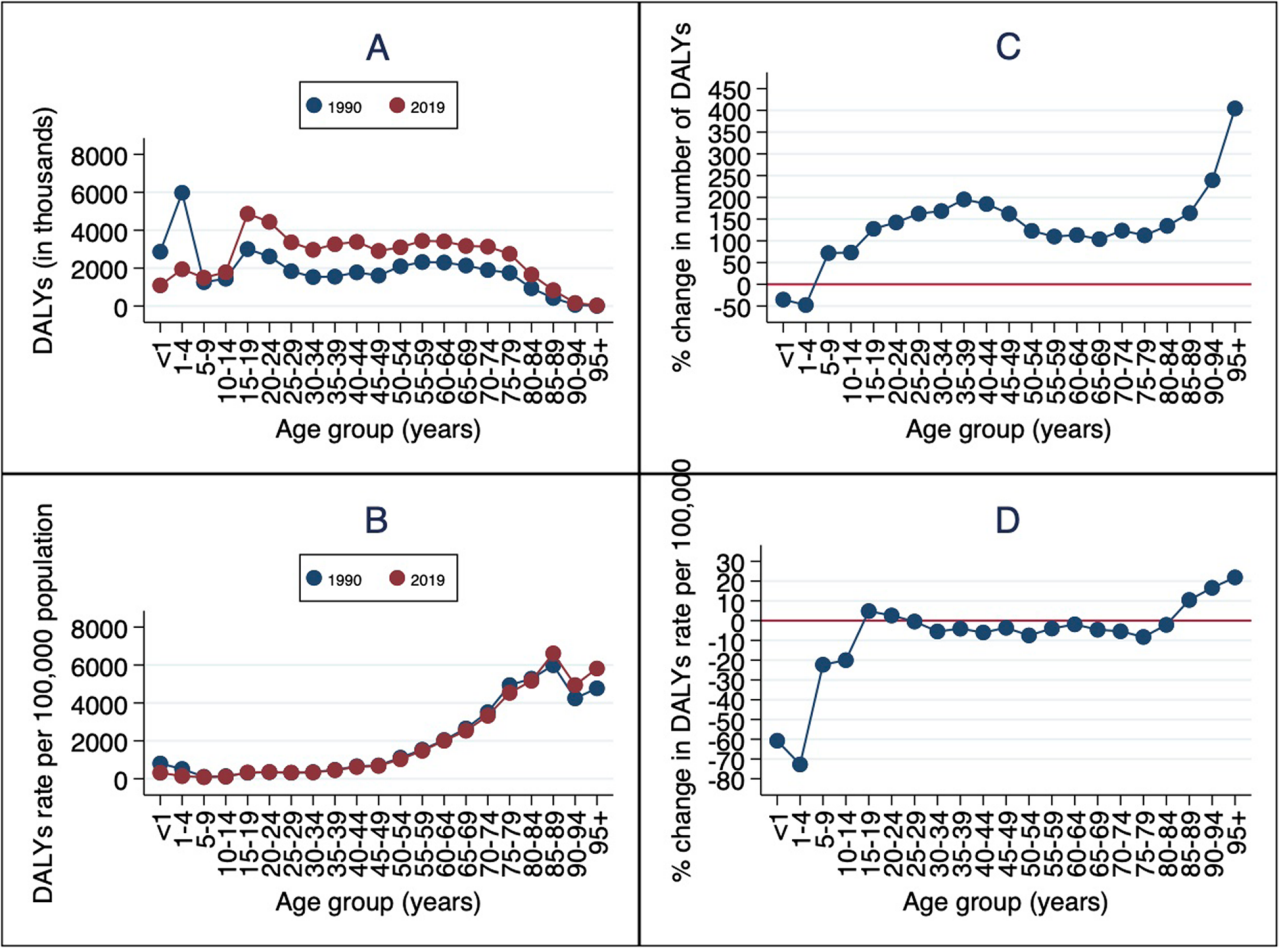
DALYs in thousands (**A**); rate per 100,000 population (**B**) of DALYs for CKD; Percent change (2019 vs 1990) in number of DALYs (**C**); Percent change (2019 vs 1990) in DALYs rate per 100,000 population (**D**). Abbreviations: DALY=disability-adjusted life-year

The number of DALYs for CKD was estimated as 76.03 million (95% UI: 61.01 to 93.36) in 2019 compared to 39.42 million (95% UI: 33.09 to 45.90) in 1990, representing 93% increase whereas the DALYs rate per 100,000 population was estimated as 416.89 (95% UI: 334.53 to 511.93) in 2019 compared to 496.38 (95% UI: 416.55 to 577.87) in 1990, representing 16% reduction. (Table 1). The number of DALYs (Fig. 2A) and percent change in number of DALYs (Fig. 2C) varies by age group. The DALYs rate per 100,000 population was highest among adults aged 85–89 years (Fig. 2B). Compared to 1990, there were increases in DALYs rate per 100,000 population among adolescent and young adults (15–24 years) and among adults (85 + years) (Fig. 2D)

**Table 1:** Number of DALYs and DALYs rate per 100,000 population of CKD with percent change in Zambia, 1990–2019

Characteristics	CKD; Number of DALYs in thousands (95%UI)							CKD; Rate per 100,000 persons (95%UI)						
	1990			2019			% change	1990			2019			% change
Sex	#	Lower	Upper	#	Lower	Upper		Rate	Lower	Upper	Rate	Lower	Upper	
Male	18,909.93	15,934.15	22,587.37	43,350.28	34,361.60	53,557.23	129.25	484.85	408.56	579.14	480.77	381.08	593.97	-0.84
Female	20,517.73	15,708.64	25,030.06	32,680.74	25,764.76	41,085.69	59.28	507.5	388.55	619.11	354.42	279.42	445.57	-30.16
Both	39,427.66	33,086.64	45,900.39	76,031.02	61,011.27	93,363.98	92.84	496.38	416.55	577.87	416.89	334.53	511.93	-16.01
Age (years)														
<1	2,865.31	1,563.31	4,361.56	1,856.10	1,090.17	3,234.51	-35.22	803.42	438.34	1,222.96	315.34	185.21	549.53	-60.75
1-4	5,977.46	2,257.37	9,089.39	3,131.57	1,942.72	5,290.50	-47.61	515.28	194.6	783.55	140.38	87.09	237.16	-72.76
5-9	1,262.79	808.34	1,758.25	2,170.65	1,493.11	3,078.93	71.89	107.89	69.06	150.22	83.84	57.67	118.92	-22.29
10-14	1,443.05	1,039.45	1,908.17	2,497.78	1,786.46	3,391.95	73.09	134.27	96.72	177.55	107.43	76.83	145.88	-19.99
15-19	3,004.32	2,179.47	3,964.79	6,839.88	4,867.39	9,391.31	127.67	317.65	230.43	419.2	332.97	236.95	457.18	4.82
20-24	2,620.15	1,874.21	3,507.89	6,349.70	4,441.20	8,758.24	142.34	348.17	249.05	466.14	357.24	249.86	492.74	2.61
25-29	1,838.21	1,369.09	2,413.33	4,827.78	3,361.63	6,679.56	162.63	322.86	240.47	423.87	321.43	223.82	444.73	-0.44
30-34	1,530.67	1,135.11	2,051.44	4,111.40	2,964.58	5,734.82	168.60	351.44	260.62	471.01	332.24	239.57	463.43	-5.46
35-39	1,551.77	1,136.78	2,027.31	4,583.68	3,259.78	6,529.10	195.38	469.83	344.18	613.81	450.81	320.6	642.14	-4.05
40-44	1,777.17	1,315.19	2,389.44	5,060.87	3,386.62	7,057.96	184.77	658.07	487.01	884.8	618.63	413.97	862.75	-5.99
45-49	1,609.20	1,219.94	2,107.53	4,220.32	2,907.24	5,943.09	162.26	704.22	533.87	922.3	678.84	467.63	955.95	-3.60
50-54	2,097.12	1,579.15	2,736.07	4,674.20	3,102.56	6,503.13	122.89	1,111.70	837.12	1,450.41	1,028.76	682.85	1,431.29	-7.46
55-59	2,322.12	1,690.93	3,070.21	4,872.35	3,440.47	6,949.45	109.82	1,535.69	1,118.26	2,030.42	1,474.37	1,041.09	2,102.90	-3.99
60-64	2,297.19	1,691.73	3,060.29	4,903.93	3,408.50	6,700.40	113.48	2,042.85	1,504.43	2,721.47	2,004.25	1,393.07	2,738.48	-1.89
65-69	2,138.32	1,583.24	2,864.45	4,357.31	3,171.14	5,841.71	103.77	2,655.48	1,966.15	3,557.22	2,534.34	1,844.43	3,397.71	-4.56
70-74	1,900.90	1,387.14	2,491.15	4,249.84	3,131.49	5,658.71	123.57	3,513.81	2,564.13	4,604.89	3,324.45	2,449.62	4,426.54	-5.39
75-79	1,750.43	1,288.64	2,344.49	3,723.44	2,755.05	4,846.97	112.72	4,938.08	3,635.33	6,613.95	4,530.72	3,352.37	5,897.84	-8.25
80-84	941.61	704.39	1,261.75	2,206.82	1,658.78	2,816.28	134.37	5,279.78	3,949.68	7,074.87	5,166.85	3,883.72	6,593.77	-2.14
85-89	424.7	313.55	568.39	1,120.81	836.79	1,466.96	163.91	5,990.30	4,422.56	8,016.93	6,615.92	4,939.41	8,659.13	10.44
90-94	64.67	47.48	85.52	219.57	163.8	281.99	239.52	4,239.13	3,111.94	5,605.73	4,942.94	3,687.56	6,348.17	16.60
95+	10.51	7.41	15.08	53.01	33.74	72.05	404.38	4,772.46	3,364.38	6,847.54	5,816.65	3,701.85	7,906.46	21.88
All ages	39,427.66	33,086.64	45,900.39	76,031.02	61,011.27	93,363.98	92.84	496.38	416.55	577.87	416.89	334.53	511.93	-16.01

*Abbreviations*: *CKD* Chronic kidney disease, *HTN *Hypertension, *UI* Uncertainty Interval

### Average annual rate of change in the burden of CKD

**Fig. 3:**
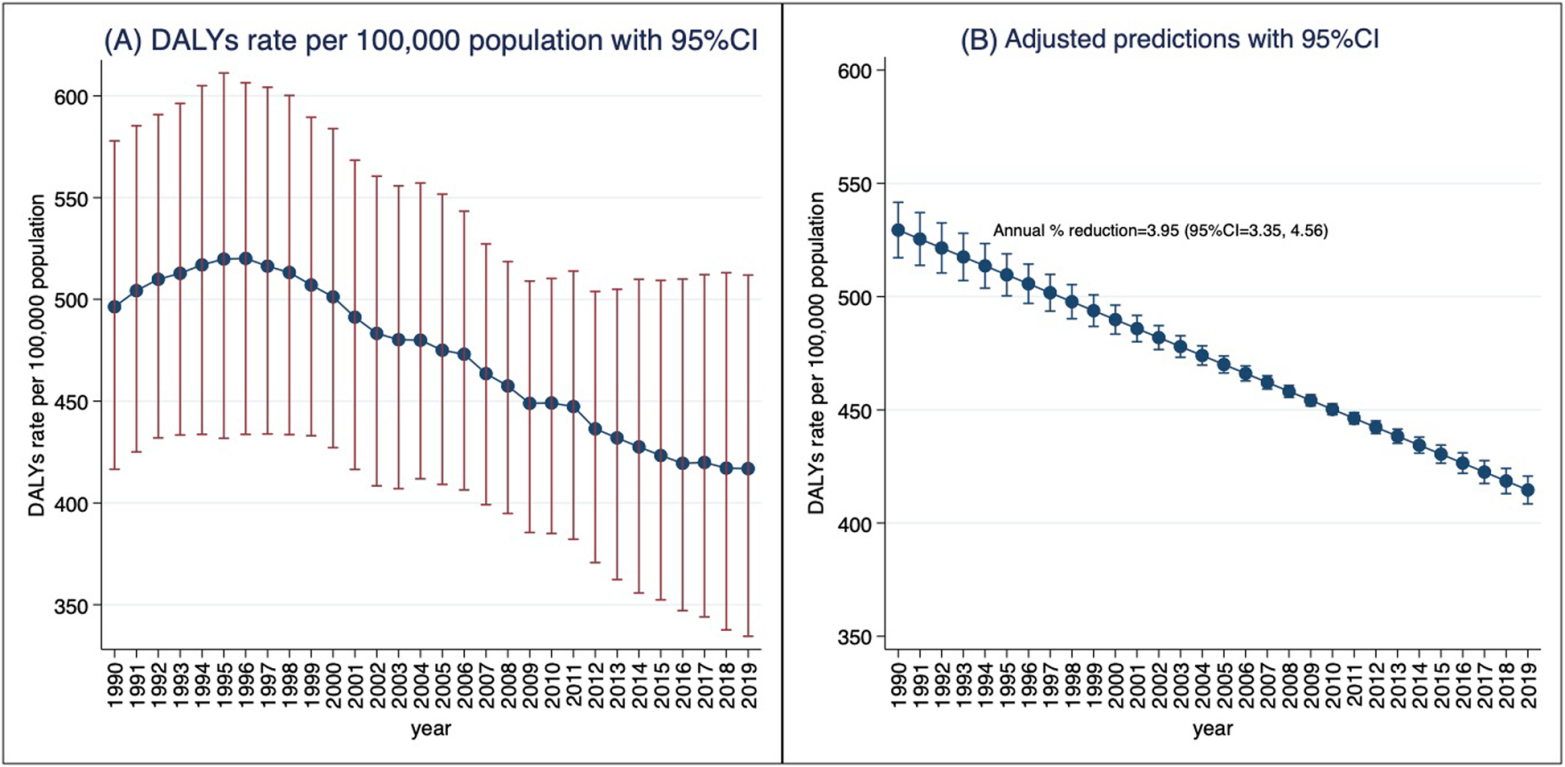
DALYs rate per 100,000 population for CKD (A) and adjusted annual predictions and percent reduction (B), 1990 to 2019, Zambia. “Annual % reduction” was estimated using linear regression model with robust standard error

DALYs rate increased from 496.38 to 100,000 population ((95% uncertainty interval (UI): 416.55 to 577.87) in 1990 to 520.11 per 100,000 population (95%UI: 433.68 to 606.40) in 1996 and decreases thereafter to 416.88 per 100,000 population (95%UI: 334.53 to 511.92) in 2019 (Fig. 3A), showing an average annual percent reduction of 3.95% (95% confidence interval (CI): 3.35, 4.56) (Fig. 3B).

### Burden of chronic kidney disease causal relations

**Fig. 4:**
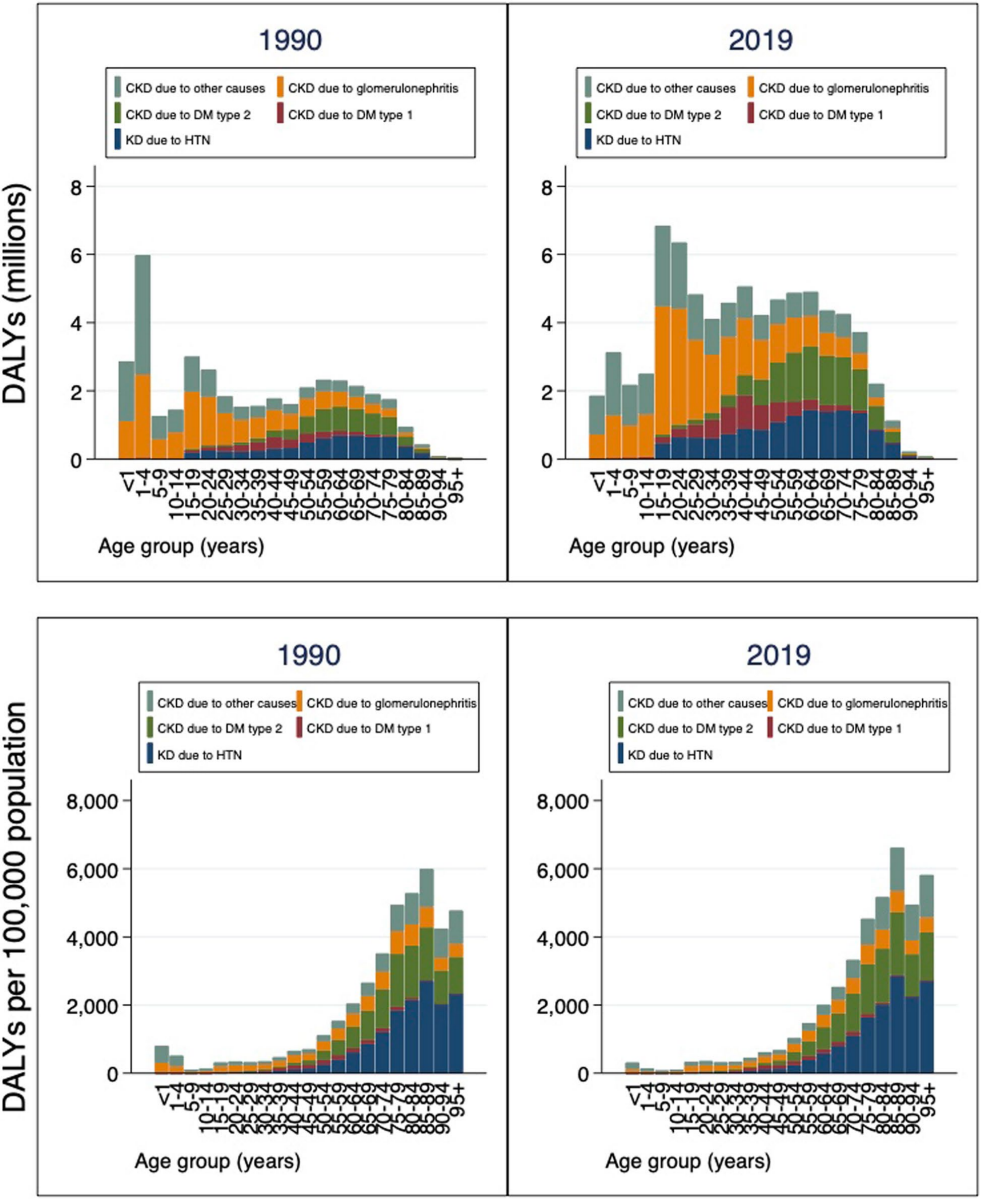
Number and rate per 100,000 population of DALYs for CKD by underlying cause, 1990-2019, Zambia. Abbreviations: DALY=disability-adjusted life-year. CKD=chronic kidney disease; HTN=Hypertension

Of the 76.03 million (95% UI: 61.01 to 93.36) CKD DALYs in 2019, about 14.25 million (95% UI: 10.70 to 18.74) were due to hypertension, 25.07 million ((95% UI: 19.01 to 32.84) due to glomerulonephritis, 5.79 million (95% UI: 3.67 to 8.46) due to diabetes mellitus type 1, 11.50 million (95% UI: 8.29 to 15.50) due to diabetes mellitus type 2, and 19.40 million ((95% UI: 14.78 to 24.61) due to other and unspecified causes. These suggest that CKD due to diabetes (types 1 and 2) accounted for 22.7% of CKD DALY while CKD due to hypertension accounted for 18.7% of CKD DALYs in 2019. The CKD due to glomerulonephritis accounted for 33.0% of CKD DALYs, the largest contribution in terms of absolute number of DALYs of any cause in 2019. In 2019, CKD due to glomerulonephritis impact adolescent (15–19 years) and young adults (20–24 years) whereas CKD due to hypertension impact adults 60 years and above (Fig. 4).

## Discussion

Zambia suffers from a substantial burden of CKD with an estimated 76.03 million DALYs and 416.89 DALYs per 100,000 population in 2019. Despite a slight annual percent reduction of 3.95% over the past two and half decades, there remains a large burden with limited access to prevention and treatment as Zambia has only one nephrologist per 2.6 million population (0.4 per million population). The International Society of Nephrology 2019 report estimates that less than 10% of the Zambian population with ESKD has access to life-sustaining therapy with dialysis [21]. Only a handful of people who have personal resources have access to life-saving therapy with kidney transplantation in other countries (e.g., India, South Africa).

Most concerning is the disparities seen in our analyses in number of DALYs for CKD by age group, with the highest burden occurring among adolescents aged 15–19 years. This is reflected as well at the young age of dialysis patients in Zambia; 66% of the Zambian dialysis population is < 50 years (compared to high income countries like the United States where < 20% of the dialysis population is < 50 years of age) [22–25]. One DALY represents the loss of the equivalent of one year of full health. The DALY is a metric that combines the burden of mortality and morbidity (non-fatal health problems) into a single number [26]. It is the primary metric used by the World Health Organization to assess the global burden of disease [8], and the primary metric used by projects to quantify the cost-effectiveness of different interventions [27]. DALYs impacting the adolescent and young adult population have far reaching ramifications: loss of investment in past education and training for young professionals, loss of future potential earnings for the family and the economy, and loss of future potential advancement that young adults suffering from CKD could contribute to society as leaders and educators in their respective fields.

Our results may under-estimate the true burden of CKD in Zambia as the GBD is reliant on published cohort, clinical trials, and model estimates. A cross-sectional survey of adults in the Kabwe District showed that up to 30% had abnormal creatinine values, suggesting undiagnosed CKD [28]. Among those with HIV, estimates suggest 28% may have CKD [29]. Among hospitalized Zambian adults with COVID-19, there were high rates of chronic conditions (hypertension in 50%, diabetes mellitus 28.5%, and CKD 8%) [30]. Among a recent retrospective cohort of critically ill adults, 50% had kidney disease and the average age was 36 years [31].

In Zambia, hypertension and diabetes are important causes of chronic kidney disease with an estimated 18.7% of CKD DALYs due to hypertension while 22.7% of CKD DALYs were due to diabetes. Estimates suggest hypertension and its subsequent end organ damage (e.g., kidney disease) may impact up to 1 in 5 people worldwide but low- middle-income countries (LMICs) face the heaviest burden [32–34]. For example, 75% of the estimated 1.4 billion persons with hypertension live in LMICs [35]. A systemic review of youth and adolescents in Africa estimate similar rates to adults with 1 in 5 impacted, yet this population is rarely screened – leading to untreated disease that progresses for years. Even though existing international hypertension guidelines have been shown effective, lack of adaptation to the Zambian setting and subsequent gaps in protocol implementation may be key drivers in poor hypertension care and resultant early-onset ESKD in Zambia [7, 36, 37]. Similar to our findings, hypertension in Africa is resulting in much younger adults needing end of life care with dialysis, compared to high-income countries (HICs) [22, 38]. Lack of screening for CKD/ESKD in hypertension and diabetes are missed opportunities for improving care as similar to hypertension, kidney disease often does not present with symptoms until very late stages. Therefore, it can progress undetected for years, or even decades, until non-pharmaceutical and simpler interventions are ineffective and advanced technology for treatment is required (i.e., dialysis, transplantation) [39, 40].

Prevalent hypertension in Zambia is 100 times the rate of cancer[41], 5 times the rate of diabetes mellitus, and twice that of prevalent HIV [42–44]. Yet, little attention has been focused on this burdensome disease [45], which impacts youth at a time when end organ effects, which are fatal in Zambia, could be prevented or delayed for decades – saving thousands if not millions of lives as is the case in HICs. Though hypertension may contribute to half of NCDs in Zambia, it is not in isolation [30, 46]. In addition to being a consequence of hypertension, kidney disease may also be a driving contributor to hypertension development.

Given the high burden of DALYs attributable to CKD in adolescents and young adults, it is imperative to address the largest contributor to CKD is glomerulonephritis, contributing to 33% of all CKD DALYs. There is limited data on glomerulonephritis in Africa; the few reports out there suggest quite poor outcomes. A systematic review (insufficient data for a meta-analysis) revealed 5-year survival from lupus nephritis ranged from 54 to 94% with worse outcomes in sub-Saharan Africa [47]. There is no specific data on epidemiology or outcomes of glomerulonephritis for Zambia. The only study published in 2007 in children reported sub-optimal care of glomerulonephritis from primary to tertiary health care facilities in Zambia showed that of the 34 children with glomerulonephritis who had been referred to UTH pediatric renal clinic from across the country, none had a renal biopsy to confirm diagnosis and guide treatment [48]. Nearly two thirds of the children did not have a working diagnosis at the time of referral with a 23% mortality post referral[48]. Although limited to a small study, the findings underscore the need for improved screening, diagnosis, and treatment of glomerulonephritis in Zambia.

If diagnosed early and treated effectively, glomerulonephritis can be managed yet access to expedient biopsy results and induction therapies are imperative. However, Zambia’s public sector has limited access to these resources. Currently kidney biopsies in both public and private sectors are processed and analyzed in India and South Africa, taking 10–14 days to get results, limiting the effectiveness of early therapies. Standard corticosteroids are typically available but steroid-sparing agents such as mycophenolate mofetil, cyclophosphamide, or rituximab are limited in their availability. Plasmapheresis is only available in two public hospitals in capital city of Lusaka.

The limitations of GBD have been discussed elsewhere [49, 50]. We have discussed those specific to our study. First, Zambia does not have high-quality population-based studies on the prevalence of CKD, particularly in children. Where data is limited, GBD rely on statistical techniques to estimate CKD burden in these regions. Second, equations for eGFR estimation vary in publications and this is an inherent limitation to GBD and this analysis. Third, most data sources reporting the prevalence of non-fatal CKD are cross-sectional and do not repeat serum creatinine and urine albumin-to-creatinine ratio (ACR) measurements over 3 months [51]. Fourth, ascertainment of the cause of CKD is difficult. Biopsy is the gold-standard method for assigning the underlying cause of CKD, but this procedure is only advised when confirmation of cause is necessary, and the benefits of confirmation outweigh risks of the procedure. Fifth, there might be possible misclassification of glomerulonephritis, which frequently are concurrent with hypertension. Consequently, this could bias towards under reporting for hypertension or over reporting of glomerulonephritis. For example, there is a chance that CKD patients who had proteinuria and body swelling were classified as glomerulonephritis when in fact these signs were due to CKD, secondary to other causes and not glomerulonephritis. Furthermore, in Zambia, the prevalence of Hepatitis B virus was estimated as 5.6% among adults aged 15–59; whereas the prevalence among HIV positive individuals was 7.1% [52]. With the high prevalence of Hepatitis in the Zambian population, it is reasonable to speculate the role of viral hepatitis on the large number of patients with glomerulonephritis. Limitations notwithstanding, our study can provide insight to further understand the disease burden of CKD and underlying causes and as a basis for setting priorities for action.

## Conclusion

The burden of CKD remains high in the Zambian population with diabetes, high blood pressure, and glomerulonephritis as important causes. Particularly important is the impact of CKD and its sequalae on the young Zambian working force. The results highlight the need to develop a comprehensive action plan to prevent and treat kidney disease, including public awareness of CKD, educational programmes for health-care personnel, early treatment of potential causes of CKD, and improved access to diagnostic and therapeutic tools that are available elsewhere. The high burden of CKD among Zambians with limited adaptation of existing international guidelines for ease of screening, prevention and treatment necessitates improved government, private, and non-profit support for kidney disease in Zambia.

## Data Availability

Data for this study were obtained from the GBD 2019 study. This study adherence to the Guidelines for Accurate and Transparent Health Estimates Reporting (GATHER) recommendations. All data used in this study were obtained from the Institute for Health Metrics and Evaluation (IHME) website: http:// ghdx. healt hdata. org/ gbd- resul ts- tool. (Accessed Aug 27, 2022) [10]. The public access to the database is open.

## Abbreviations

GBD: Global Burden of Disease
CKD: Chronic kidney disease
SSA: Sub-Saharan Africa
YLD: Years lived with disability
YLL: Years of life lost
DALY: Disability-adjusted life-years
QALY: quality-adjusted life year
UI: Uncertainty interval
ESKD: End-stage kidney disease
COVID-19: Coronavirus disease 2019
eGFR: estimated glomerular filtration rate
ACR: Albumin-to-creatinine ratio
UTH: University Teaching Hospital
HICs: high-income countries
HIV: human immunodeficiency virus
CRA: comparative risk assessment
GATHER: Guidelines for Accurate and Transparent Health Estimates Reporting
HTN: Hypertension
LMICs: low- middle-income countries
NCDs: Non-Communicable Diseases
